# Dietary Polyphenol Intake is Associated with HDL-Cholesterol and A Better Profile of other Components of the Metabolic Syndrome: A PREDIMED-Plus Sub-Study

**DOI:** 10.3390/nu12030689

**Published:** 2020-03-04

**Authors:** Sara Castro-Barquero, Anna Tresserra-Rimbau, Facundo Vitelli-Storelli, Mónica Doménech, Jordi Salas-Salvadó, Vicente Martín-Sánchez, María Rubín-García, Pilar Buil-Cosiales, Dolores Corella, Montserrat Fitó, Dora Romaguera, Jesús Vioque, Ángel María Alonso-Gómez, Julia Wärnberg, José Alfredo Martínez, Luís Serra-Majem, Francisco José Tinahones, José Lapetra, Xavier Pintó, Josep Antonio Tur, Antonio Garcia-Rios, Laura García-Molina, Miguel Delgado-Rodriguez, Pilar Matía-Martín, Lidia Daimiel, Josep Vidal, Clotilde Vázquez, Montserrat Cofán, Andrea Romanos-Nanclares, Nerea Becerra-Tomas, Rocio Barragan, Olga Castañer, Jadwiga Konieczna, Sandra González-Palacios, Carolina Sorto-Sánchez, Jessica Pérez-López, María Angeles Zulet, Inmaculada Bautista-Castaño, Rosa Casas, Ana María Gómez-Perez, José Manuel Santos-Lozano, María Ángeles Rodríguez-Sanchez, Alicia Julibert, Nerea Martín-Calvo, Pablo Hernández-Alonso, José V Sorlí, Albert Sanllorente, Aina María Galmés-Panadés, Eugenio Cases-Pérez, Leire Goicolea-Güemez, Miguel Ruiz-Canela, Nancy Babio, Álvaro Hernáez, Rosa María Lamuela-Raventós, Ramon Estruch

**Affiliations:** 1 Department of Medicine, Faculty of Medicine and Life Sciences, University of Barcelona, Barcelona, Spain. Institut d’Investigacions Biomèdiques August Pi I Sunyer (IDIBAPS), 08036 Barcelona, Spain; sara.castro@ub.edu (S.C.-B.); mdomen@clinic.cat (M.D.); rcasas1@clinic.cat (R.C.); alvaro.hernaez1@gmail.com (Á.H.); 2 Centro de Investigación Biomédica en Red Fisiopatología de la Obesidad y la Nutrición (CIBEROBN), Instituto de Salud Carlos III, 28029 Madrid, Spainjordi.salas@urv.cat (J.S.-S.); pilarbuilc@gmail.com (P.B.-C.); dolores.corella@uv.es (D.C.); mfito@imim.es (M.F.); mariaadoracion.romaguera@ssib.es (D.R.); angelmaria.alonsogomez@osakidetza.eus (Á.M.A.-G.); jwarnberg@uma.es (J.W.); jalfmtz@unav.es (J.A.M.); lluis.serra@ulpgc.es (L.S.-M.); fjtinahones@uma.es (F.J.T.); jose.lapetra.sspa@juntadeandalucia.es (J.L.); xpinto@bellvitgehospital.cat (X.P.); pep.tur@uib.es (J.A.T.); clotilde.vazquez@fjd.es (C.V.); mcofan@clinic.cat (M.C.); nerea.becerra@urv.cat (N.B.-T.); rocio.barragan@uv.es (R.B.); ocastaner@imim.es (O.C.); jadwiga.konieczna@ssib.es (J.K.); daisysorto2@yahoo.com (C.S.-S.); jessicaperezlopez@uma.es (J.P.-L.); mazulet@unav.es (M.A.Z.); inmaculada.bautista@ulpgc.es (I.B.-C.); anamgp86@gmail.com (A.M.G.-P.); josem.santos.lozano.sspa@juntadeandalucia.es (J.M.S.-L.); alicia.julibert@uib.es (A.J.); nmartincalvo@unav.es (N.M.-C.); pablo1280@gmail.com (P.H.-A.); Sorli@uv.es (J.V.S.); albertsanllorente@gmail.com (A.S.); aina.galmes@uib.es (A.M.G.-P.); leiregoiko@gmail.com (L.G.-G.); mcanela@unav.es (M.R.-C.); nancy.babio@urv.cat (N.B.); lamuela@ub.edu (R.M.L.-R.); 3 Universitat Rovira i Virgili, Departament de Bioquímica i Biotecnologia, Unitat de Nutrició, 43204 Reus, Spain; 4 University Hospital of Sant Joan de Reus, Nutrition Unit, 43201 Reus, Spain; 5 Institut d’Investigació Sanitària Pere Virgili (IISPV), 43201 Reus, Spain; 6 Institute of Biomedicine (IBIOMED), University of León, 24071 León, Spain; fvits@unileon.es (F.V.-S.); vicente.martin@unileon.es (V.M.-S.); mrubig02@estudiantes.unileon.es (M.R.-G.); 7 CIBER de Epidemiología y Salud Pública (CIBERESP), Instituto de Salud Carlos III, 28029 Madrid, Spain; vioque@umh.es (J.V.); lgarmol@ugr.es (L.G.-M.); sandra.gonzalezp@umh.es (S.G.-P.); 8 University of Navarra, Department of Preventive Medicine and Public Health, Instituto de Investigación Sanitaria de Navarra (IdiSNA), 31008 Pamplona, Spain; aromanos@unav.es; 9 Servicio Navarro de Salud-Osasunbidea-Instituto de Investigación Sanitaria de Navarra (IdiSNA), 31008 Pamplona, Spain; 10 Department of Preventive Medicine, University of Valencia, 46010 Valencia, Spain; 11 Cardiovascular Risk and Nutrition Research group, Institut Hospital del Mar de Investigaciones Médicas (IMIM), 08007 Barcelona, Spain; 12 Health Research Institute of the Balearic Islands (IdISBa), University Hospital Son Espases (Research Unit), 07120 Palma de Mallorca, Spain; 13 Miguel Hernandez University, ISABIAL-FISABIO, 03010 Alicante, Spain; mdelgado@ujaen.es; 14 Bioaraba Health Research Institute; Osakidetza Basque Health Service, Araba University Hospital; University of the Basque Country UPV/EHU, 01009 Vitoria-Gasteiz, Spain; 15 Department of Nursing. University of Málaga, Instituto de Investigación Biomédica de Málaga (IBIMA), 29010 Málaga, Spain; 16 Department of Nutrition, Food Sciences, and Physiology, Center for Nutrition Research, University of Navarra, 31008 Pamplona, Spain; 17 Precision Nutrition Program, IMDEA Food, CEI UAM + CSIC, 28049 Madrid, Spain; lidia.daimiel@imdea.org; 18 Research Institute of Biomedical and Health Sciences (IUIBS), University of Las Palmas de Gran Canaria & Centro Hospitalario Universitario Insular Materno Infantil (CHUIMI), Canarian Health Service, 35016 Las Palmas de Gran Canaria, Spain; 19 Virgen de la Victoria Hospital, Department of Endocrinology, Instituto de Investigación Biomédica de Málaga (IBIMA). University of Málaga, 29010 Málaga, Spain; 20 Department of Family Medicine, Research Unit, Distrito Sanitario Atención Primaria Sevilla, 41010 Sevilla, Spain; 21 Lipids and Vascular Risk Unit, Internal Medicine, Hospital Universitario de Bellvitge, IDIBELL, Hospitalet de Llobregat, 08908 Barcelona, Spain; mrodriguezsa@bellvitgehospital.cat; 22 Research Group on Community Nutrition & Oxidative Stress, University of Balearic Islands, 07122 Palma de Mallorca, Spain; 23 Department of Internal Medicine, Maimonides Biomedical Research Institute of Cordoba (IMIBIC), Reina Sofia University Hospital, University of Cordoba, 14004 Cordoba, Spain; angarios2004@yahoo.es; 24 Department of Preventive Medicine and Public Health, University of Granada, 18016 Granada, Spain; 25 Division of Preventive Medicine, Faculty of Medicine, University of Jaén, 23071 Jaén, Spain; 26 Department of Endocrinology and Nutrition, Instituto de Investigación Sanitaria Hospital Clínico San Carlos (IdISSC), 28040 Madrid, Spain; mmatia@ucm.es; 27 CIBER Diabetes y Enfermedades Metabólicas (CIBERDEM), Instituto de Salud Carlos III (ISCIII), 28029 Madrid, Spain; jovidal@clinic.cat; 28 Department of Endocrinology, Institut d’Investigacions Biomédiques August Pi Sunyer (IDIBAPS), Hospital Clinic, University of Barcelona, 08036 Barcelona, Spain; 29 Department of Endocrinology and Nutrition, Hospital Fundación Jimenez Díaz, Instituto de Investigaciones Biomédicas IISFJD. University Autonoma, 28040 Madrid, Spain; 30 Lipid Clinic, Department of Endocrinology and Nutrition, Institut d’Investigacions Biomèdiques August Pi Sunyer (IDIBAPS), Hospital Clínic, 08036 Barcelona, Spain; 31 Unidad de Gestión Clínica de Endocrinología y Nutrición del Hospital Virgen de la Victoria, Instituto de Investigación Biomédica de Málaga (IBIMA), 29010 Málaga, Spain; 32 Centro de Salud Raval, 03203 Alicante, Spain; ecases@coma.es; 33 Department of Nutrition, Food Science and Gastronomy, XaRTA, INSA, School of Pharmacy and Food Sciences, University of Barcelona, 08028 Barcelona, Spain; 34 Department of Internal Medicine, Hospital Clinic de Barcelona, 08036 Barcelona, Spain

**Keywords:** polyphenols, metabolic syndrome, Mediterranean diet, glignans, stilbenes, HDL-cholesterol

## Abstract

Dietary polyphenol intake is associated with improvement of metabolic disturbances. The aims of the present study are to describe dietary polyphenol intake in a population with metabolic syndrome (MetS) and to examine the association between polyphenol intake and the components of MetS. This cross-sectional analysis involved 6633 men and women included in the PREDIMED (PREvención con DIeta MEDiterranea-Plus) study. The polyphenol content of foods was estimated from the Phenol-Explorer 3.6 database. The mean of total polyphenol intake was 846 ± 318 mg/day. Except for stilbenes, women had higher polyphenol intake than men. Total polyphenol intake was higher in older participants (>70 years of age) compared to their younger counterparts. Participants with body mass index (BMI) >35 kg/m^2^ reported lower total polyphenol, flavonoid, and stilbene intake than those with lower BMI. Total polyphenol intake was not associated with a better profile concerning MetS components, except for high-density lipoprotein cholesterol (HDL-c), although stilbenes, lignans, and other polyphenols showed an inverse association with blood pressure, fasting plasma glucose, and triglycerides. A direct association with HDL-c was found for all subclasses except lignans and phenolic acids. To conclude, in participants with MetS, higher intake of several polyphenol subclasses was associated with a better profile of MetS components, especially HDL-c.

## 1. Introduction

Polyphenols are plant-derived molecules characterized by the presence of one or more aromatic rings and attached hydroxyl groups [1]. They are classified into five subclasses according to their chemical structure, including flavonoids and nonflavonoids subclasses defined as phenolic acids, stilbenes, lignans, and other polyphenols. These bioactive compounds are responsible for some health and sensory properties of foods, such as bitterness, astringency, and antioxidant capacity. The intake of phenolic compounds and their food sources is highly variable and depends on dietary patterns, sex, socioeconomic factors, and the native foods of each region [2]. The Mediterranean diet (MedDiet) is characterized by a high intake of phenolic compounds because MedDiet interventions promote the intake of phenolic rich and plant-based products, such as legumes, vegetables, fruits, nuts and wholegrain cereals, and promote the use of extra virgin olive oil as the main source of fat. It has been suggested that phenolic compounds are partly responsible for the beneficial effects attributed to the MedDiet [3].

The metabolic syndrome (MetS) is defined as a cluster of metabolic disturbances, which include impaired glucose metabolism, elevated blood pressure, and low level of HDL-c, dyslipidemia, and abdominal obesity [4]. Sedentary lifestyle, smoking, and unbalanced diets are well-known modifiable risk factors for MetS, and lifestyle interventions in those areas, especially dietary interventions based on the MedDiet [3,4,5,6], might improve this condition. Considering the chronic low-grade inflammation and oxidative stress observed in MetS, polyphenols are good candidates to improve the condition because of their antioxidant and anti-inflammatory properties [7]. Moreover, several epidemiological studies have observed a negative association between polyphenol intake and MetS rates [8]. Regarding MetS components, an adequate intake of phenolic compounds has been shown to improve lipid profile and insulin resistance, and decrease blood pressure levels and body weight [8,9].

Despite the fact that phenol-rich dietary patterns are effective in improving some MetS components, there is no single phenolic compound or extract able to improve all the components of MetS [10]. Nevertheless, given the complexity of MetS and the heterogeneity of polyphenols, more large randomized trials with MetS patients are needed to evaluate the effect of polyphenol intake in reducing MetS complications, and whether intake of the different polyphenol subclasses could be associated with improvements in MetS components, because each subtype has different absorption and metabolism [11].

Therefore, the aims of our study were firstly to describe polyphenol intake in 6633 participants with MetS from the PREvención con DIeta MEDiterranea-Plus (PREDIMED-Plus) trial and to identify the main food sources of polyphenols in those participants, and secondly to examine whether higher intakes of some polyphenol sub-classes are associated with MetS components in this population.

## 2. Materials and Methods

### 2.1. Design of the Study

A cross-sectional analysis of the baseline data of participants included in the PREvención con DIeta MEDiterranea-Plus (PREDIMED-Plus) study was performed. The profile of the cohort, recruiting methods, and data collection processes have been described elsewhere [12] and on the website http://predimedplus.com. The study protocol was approved by the 23 recruiting centers Institutional Review Boards and registered in 2014 at the International Standard Randomized Controlled Trial Number registry (http://www.isrctn.com/ISRCTN89898870). All participants provided written informed consent before joining the study.

### 2.2. Participants

**Figure 1:**
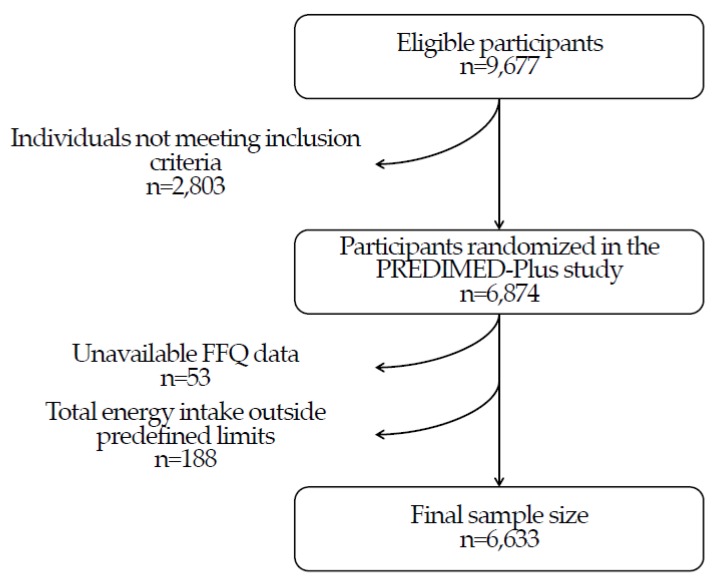
Flowchart of the participants.

A total of 6874 subjects were recruited and randomized in the 23 recruiting centers between September 2013 and December 2016. Primary care medical doctors from primary care centers of the National Health System assessed potential participants for eligibility. Eligible participants were men (aged 55–75 years) and women (aged 60–75 years) with overweight or obesity (body mass index [BMI] ≥27 and <40 kg/m^2^) and at least three components of MetS according to the comprehensive definition of the International Diabetes Federation; National Heart, Lung, and Blood Institute; and American Heart Association (2009) [4]. Exclusion criteria were documented history of cardiovascular diseases (CVD), having a long-term illness, drug or alcohol use disorder, a BMI of 40 or higher, a history of allergy or intolerance to extra virgin olive oil or nuts, malignant cancer, inability to follow the recommended diet or physical activity program, history of surgical procedures for weight loss, and obesity of known endocrine disease (except for treated hypothyroidism). Of the total sample of 6874 randomized participants, 241 participants were excluded from the current analysis (Figure 1): 53 without food-frequency questionnaire (FFQ) data at baseline, and 188 participants who reported energy intake values outside the predefined limits (<3347 kJ [800 kcal]/day or >17,573 kJ [4000 kcal]/day for men; <2510 kJ [500 kcal]/day or >14,644 kJ [3500 kcal]/day for women) [13].

### 2.3. Estimation of Dietary Polyphenol Intake

The total dietary polyphenol intake and polyphenol subclasses were obtained at baseline by the 143-item FFQs used in the PREDIMED-Plus study. As described elsewhere [14], dietary polyphenol intake was estimated following these steps: (1) All foods from the FFQ with no polyphenol content, or only traces, were excluded; (2) recipes were calculated according to their ingredients and portions using traditional MedDiet recipes; (3) when an item from the FFQ included several foods (e.g., oranges and tangerines), the proportion of intake was calculated according to data available in the national survey; (4) no retention or yield factors were used to correct weight changes during cooking because this was already taken into account in the FFQ; (5) the polyphenol content in 100 g of each food item was obtained from the Phenol-Explorer database (version 3.6) [15]; (6) finally, the individual polyphenol intake from each food was calculated by multiplying the content of each polyphenol by the daily consumption of each food. Total polyphenol intake was calculated as the sum of all individual polyphenol intakes from the food sources reported in the FFQ.

The data used to calculate polyphenol intake was obtained by chromatography of all the phenolic compounds, except proanthocyanidins, the content of which was obtained by normal-phase high-performance liquid chromatography. In the case of lignans and phenolic acids in certain foods (i.e., swiss chard, chickpeas, plums, and strawberry jam), data corresponding to chromatography after hydrolysis was also collected, since these treatments are needed to release phenolic compounds that could otherwise not be analyzed. Total and polyphenol subclass intakes were adjusted for energy intake (kcal/day) using the residual method [13].

### 2.4. Measurements and Outcome Assessment

Data on age, sex, educational levels, anthropometric measurements, dietary habits and lifestyle were collected at baseline. Anthropometric measurements were measured according to the PREDIMED-Plus protocol. Weight was recorded with participants in light clothing without shoes or accessories using a high-quality calibrated scale. Height was measured with a wall-mounted stadiometer. Waist circumference was measured midway between the lowest rib and the iliac crest. The BMI was calculated as weight (kg) divided by the square of height (m^2^).

Physical activity and sedentary behaviors were evaluated using the validated Regicor Short Physical Activity Questionnaire [16] and the validated Spanish version of the Nurses’ Health Study questionnaire [17], respectively.

Information related to sociodemographic and lifestyle habits, individual and family medical history, smoking status, medical conditions, and medication use was evaluated using self-reported questionnaires. Sociodemographic and lifestyle variables were categorized as follows: age (three categories: <65, 65–70, or >70 years), educational level (three categories: primary, secondary, or high school), physical activity level (three categories: low, moderate, or high), BMI (three categories: 27.0–29.9, 30.0–34.9, or ≥35 kg/m^2^), and smoking status (three categories: never, former, or current smoker).

Blood samples were collected after overnight fasting. Biochemical analyses were performed to determine plasma glucose (mg/dL), glycated hemoglobin (%), HDL-c (mg/dL), and triglyceride (mg/dL) levels using standard laboratory enzymatic methods. Low-density lipoprotein cholesterol (LDL-c; mg/dL) was calculated using the Friedewald formula whenever triglyceride levels were less than 300 mg/dL. Blood pressure measurements were obtained after the participant had rested for five minutes. Each measurement was obtained with a validated semiautomatic oscillometer (Omron HEM-705CP), ensuring the use of the proper cuff size for each participant.

### 2.5. Statistical Analysis

Descriptive statistics were used to define the baseline characteristics of the participants. The database used was the PREDIMED-Plus baseline database generated in September 2018. Continuous variables are expressed as mean ± SD. Categorical variables are expressed as number (*n*) and percentage (%). Comparisons among quartiles of dietary polyphenol intake used the Pearson chi square test (χ^2^) for categorical variables or one-way ANOVA for continuous variables. The associations between dietary polyphenol intake and MetS components were analyzed by linear regression models to determine differences between quartiles of polyphenol subclass intake. The results of the regression models are expressed as unstandardized β-coefficients. For regression models, polyphenol and polyphenol subclasses are expressed as quartiles of energy-adjusted dietary intake. We used robust variance estimators to account for intra-cluster correlation in all linear models, considering members of the same household as a cluster. All regression models were adjusted for potential confounders. Model 1 was adjusted for sex, age, recruiting center, and members of the same household. Model 2 was additionally adjusted for physical activity level, BMI (except for waist circumference criteria), smoking status, and educational level. We additionally adjusted for anti-diabetic treatment when assessing glycemia and antihypertensive treatments when assessing blood pressure. Lastly, model 3 was additionally adjusted for total energy intake (continuous, kcal/day), saturated fatty acids (g/day), and distilled drinks alcohol intake (g/day). In model 3, the analysis of glycemia was additionally adjusted for dietary simple sugar intake (g/day), whereas the analysis of systolic and diastolic blood pressures was also adjusted for dietary sodium intake (mg/day). The normality of the continuous outcomes and standardized residuals was assessed with the Shapiro–Wilk test. Values are shown as 95% confidence interval (CI) and significance for all statistical tests was based on bilateral contrast set at *p* < 0.05. The P value for linear trends was computed by fitting a continuous variable that assigned the median value for each quartile in regression models. The descriptive analyses shown in Table 1, Table 2 and Table 3 were performed using SPSS software version 22.0 (Chicago, IL, USA) and the regression analysis was performed using Stata software version 16 (StataCorp LP, College Station, TX, USA).

## 3. Results

The present study was conducted on 6633 participants from the PREDIMED-Plus study. The mean age was 65.0 ± 4.9 years, and mean BMI was 32.5 ± 3.44 kg/m^2^. Table 1 shows the main characteristics of the participants according to quartiles of dietary total polyphenol intake. We observed that participants included in the highest quartile of polyphenol intake (>1019.3 mg/day) were mainly men and former smokers with a higher educational level (all three *p* < 0.001). We observed an inverse trend in the relationship between polyphenol intake and BMI (*p* = 0.02), whereas this trend was direct for waist circumference (*p* = 0.01) and body weight (*p* < 0.001). Moreover, fewer participants with insulin and nonsteroidal anti-inflammatory drug treatment were observed in the highest quartile of polyphenol intake (both *p* = 0.01).

Total polyphenol intake was 846 ± 318 mg/day, of which 58.0% were flavonoids (491 ± 253 mg/day), 33.1% phenolic acids (280 ± 131 mg/day), and the rest other polyphenols, stilbenes, and lignans (70.8 ± 41.5, 2.13 ± 3.92, and 1.53 ± 0.56 mg/day, respectively). The mean of the total polyphenol aglycone intake was 620.9 ± 273.5 mg/day. Table 2 shows the contribution (%) of each polyphenol subclass and polyphenol aglycones. The highest contributor to total polyphenol intake was hydroxycinnamic acids (30.9%). Regarding flavonoids, flavanols were the main contributors (24.1% from proanthocyanidins, 3.32% catechins, and 0.08% of theaflavins), followed by flavanones (9.83%), flavones (8.65%), flavonols (6.40%), and anthocyanins (5.14%). Additionally, tyrosols represented 6.19% of the total polyphenol intake, being the most abundant polyphenol classified within the group of other polyphenols.

The main food sources for each polyphenol subclass are also shown in Table 2. In the case of flavonoids, the most important contributors to the intake of proanthocyanidins were fruits and chocolate and its derivatives. Fruits (mainly oranges and orange juice) were the greatest contributors of flavanones, while vegetables (mainly onion, spinach, and lettuce) were the greatest contributors of flavones. Red wine, olives, tea, and wholegrain cereals were also important contributors to the remaining subclasses. Coffee was the most significant contributor of phenolic acids, especially of hydroxycinnamic acids, followed by olives and red wine. Stilbenes were mainly provided by red wine (91.9%). Lignans were widely distributed among foods, with extra virgin olive oil, fruits, and vegetables the main contributors. The main contributors of other polyphenols were olives, olive oil, cereals, coffee, and alcoholic beverages (mainly beer and red wine).

Table 3 shows the energy-adjusted intake of total polyphenols and the main subclasses by sex, age, BMI, level of physical activity, educational level, and smoking status. Total polyphenol intake was significantly higher in women due to their high intake of flavonoids (*p* < 0.001), whereas men consumed more phenolic acids (*p* = 0.003), stilbenes, and other polyphenols. The intake of total polyphenols, flavonoids, and lignans increased with age (*p* = 0.002, *p* < 0.001, and *p* = 0.006, respectively). Interestingly, participants with the highest BMI (>35 kg/m^2^) showed the lowest total polyphenol (*p* = 0.042), flavonoid (*p* = 0.004), and stilbene intake (*p* < 0.001), whereas phenolic acid intake was significantly higher in this group (*p* = 0.006). The level of physical activity was directly associated with total polyphenol intake (*p* < 0.001) and with all polyphenol classes except for phenolic acids (*p* < 0.001 in all cases except *p* = 0.03 for other polyphenols). Participants with a higher educational level (high school) showed higher total polyphenol, flavonoid, and stilbene intake (*p* < 0.001 in all cases). Current smokers reported a significantly higher intake of coffee than non-smokers (*p* < 0.001) and, consequently, showed a significantly higher intake of phenolic acids (*p* < 0.001). Otherwise, the smokers group showed significantly lower intake of flavonoids and lignans than their counterparts (*p* < 0.001, both).

**Figure 2:**
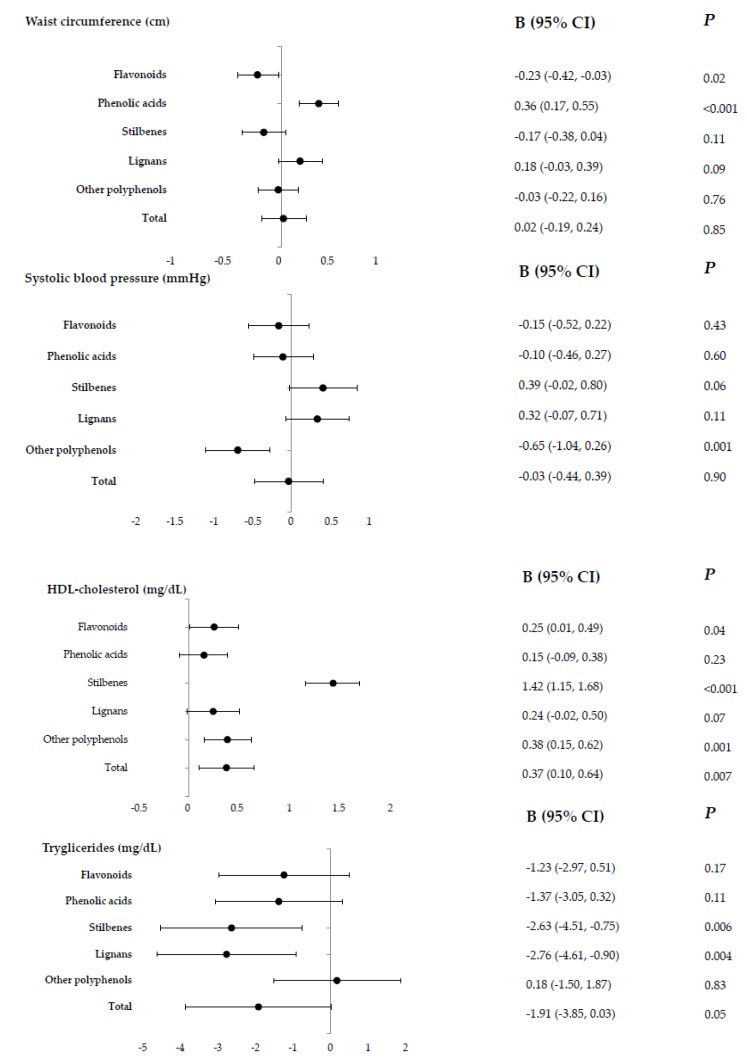
Energy-adjusted subclasses of dietary polyphenol intake by metabolic syndrome components (standardized β-coefficients [95% Confidence Intervals]).

The associations between dietary polyphenol intake and MetS components after full adjustment are shown in Figure 2. High flavonoid and low phenolic acid intake were associated with lower waist circumference (*p* = 0.02 and *p* < 0.001, respectively). The highest intake of other polyphenols was significantly and inversely associated with systolic (*p* = 0.001) and diastolic blood pressure levels (*p* = 0.002). An inverse association was found between fasting plasma glucose levels and lignans (*p* = 0.04). Positive associations were found between HDL-c levels and all polyphenol classes except for phenolic acid and lignan intake. Lastly, triglyceride concentration was inversely associated with lignans and stilbenes (*p* = 0.006 and *p* = 0.004, respectively). Changes in the linear regression models after adjustment are shown in the Supplementary Table (Supplementary Table S1).

## 4. Discussion

In this cross-sectional study of the PREDIMED-Plus study, we showed that high intake of some polyphenol subclasses was inversely associated with levels of the MetS components. These associations were especially observed for the subclasses whose contribution to total polyphenol intake was lower, such as other polyphenols, lignans, and stilbenes. Previous epidemiological studies have investigated the association between dietary polyphenol intake and MetS components in healthy populations or those at high risk of CVD, but to our knowledge there are no previous studies on these associations in subjects previously diagnosed with MetS.

In our study, the polyphenol intake was 846 ± 318 mg/day, and the intake was highest for flavonoids (58% of total), followed by phenolic acids (33.1%), similar to results of other Spanish cohorts [14,18]. By contrast, the total polyphenol intake was considerably lower than the intake observed in Mediterranean countries of the EPIC Study (1011 mg/day) [19], the SU.VI.MAX cohort study (1193 mg/day) [20], and the data from other studies conducted in non-Mediterranean countries, such as the UK National Diet and Nutrition Survey Rolling Programme for participants of similar age (1053 mg/day) [21]. The main noteworthy difference between our results and those of other countries was the relevant contribution of seeds, olives and olive oil, and red wine [14,20], while coffee, tea, and cocoa products are the foods most commonly observed in non-Mediterranean countries [22,23,24].

In addition to the differences observed according to geographical location and dietary habits, sociodemographic and lifestyle habits significantly influence the quantity and profile of intake of polyphenol subclasses. The intake of total polyphenols, particularly flavonoids and lignans, increased with age compared to younger participants (<65 years), although Grosso et al. reported the opposite observation [23]. In addition, BMI was inversely associated with total polyphenol intake, mainly with lower flavonoid and stilbene intake. This finding was also reported in the TOSCA.IT and EPIC studies [19,25].

The intake of polyphenol subclasses has been reported to have an impact on MetS components [26,27]. Even though flavonoids were the principal contributors of total polyphenol intake in our study, no associations were found with any of the MetS components, except for an inverse association with waist circumference. Similar findings were described in the HELENA study [28], where flavonoid intake was associated with lower BMI. Research on the mechanisms of action involved in the anti-obesogenic properties of flavonoids suggests that the improvements in glucose homeostasis are promoted by reducing insulin resistance and decreasing oxidative stress levels [29]. Phenolic acid intake was associated with higher fasting plasma glucose levels and waist circumference. These results are opposite from those observed in the HAPIEE cohort study, which described the beneficial effects of phenolic acid on the overall risk of developing MetS and lowering blood pressure [30]. Nevertheless, it must be taken into account that the dietary intake of phenolic acids and total polyphenol in the mentioned study doubled the amount estimated in our results, probably because of the higher intake of tea and its contribution to phenolic acid intake compared to our study population [23]. In Mediterranean countries, dietary intake of stilbenes is relatively high compared to other countries [19], with red wine being their main source (>90%). In this setting, higher stilbene intake was associated with higher HDL-c levels, but since HDL-c is the best-established cardiovascular protective factor by alcohol consumption, we cannot exclude that the alcohol content of red wine may interfere with this result [31]. In the PREDIMED study, the intake of red wine was associated with improvements in four out of five MetS criteria (i.e., elevated abdominal obesity, low HDL-c levels, high blood pressure, and high fasting plasma glucose levels) [32]. Other studies also found an inverse association between abdominal adiposity and stilbene intake, BMI, and waist circumference [30,33]. As a mechanistic pathway for stilbenes, resveratrol has shown potential anti-obesogenic effects decreasing adipocyte proliferation while activating lipolysis and β-oxidation [34]. However, the association with lower body weight and waist circumference observed in the present study and the promising effects against obesity associated with polyphenol intake observed in other studies were not clinically relevant [35]. Our results showed an inverse association between fasting glucose and lignans, and an increase in HDL-c levels and lower levels of systolic and diastolic blood pressure measurements for other polyphenols. The same finding was described in a Brazilian cohort for hypertension and other polyphenols [36]. In contrast with our results, flavonoids, mainly anthocyanins, showed greater antihypertensive effects in another study [37]. Finally, the association between lignan intake and fasting glucose levels was not demonstrated to be linked with the diagnosis of type 2 diabetes (T2D) in the EPIC study [38], but this inverse association aligned with the results observed in the PREDIMED cohort and PREDIMED-Plus study [39,40]. The potential mechanism of action underlying this association might be explained by the improvements observed in gut microbiota. This assumption was also observed in a study of US women [41], showing an inverse association between levels of gut microbiota metabolites from dietary lignan intake and T2D incidence.

Interestingly, in our study we found an association between intake of all polyphenol subclasses except phenolic acids and lignans and higher HDL-c levels. These associations were also found with total polyphenol intake in the TOSCA.IT study in T2D subjects [25] and in a similar cohort of participants at high cardiovascular risk [42]. We also observed that triglyceride levels were inversely associated with stilbene and lignan intake. Despite the fact that the antioxidant properties of polyphenols for the prevention of LDL-c oxidation are well described, the effects of dietary polyphenols on the reduction of total cholesterol levels or triglycerides are controverted [43].

The major strengths of the present study are its large sample size, the multicenter design, and the use of the Phenol-Explorer as the most comprehensive food composition database on dietary polyphenols [15]. In prior studies, the FFQ was validated to evaluate total polyphenol intake in both clinical and cross-sectional studies [44]. Our study has also some limitations. First, it used a cross-sectional design which does not allow attributing conclusions to plausible causes. In order to establish causality, a randomized controlled trial based on the intake of different polyphenol subclasses should be performed. Second, potential residual confounding and the lack of generalizability of the results to other populations than middle-aged to elderly people with higher BMI and MetS are limitations. Third, the use of the FFQ may have led to a misclassification of the exposure due to self-reported information of food intake and to the fact that some polyphenol-rich foods are grouped in the same item (e.g., spices). Nevertheless, the FFQ used has been validated in the adult Spanish population and showed good reproducibility and validity [45]. Fourth, other factors that affect food polyphenol content, such as bioavailability, variety, ripeness, culinary technique, storage, region, and environmental conditions, were not collected.

Even though recent research postulates that polyphenols are effective in improving MetS, no single phenolic compound or food has an impact on all the MetS components, suggesting that healthy and polyphenol-rich dietary patterns such as the MedDiet may be an adequate strategy for MetS management. This research might be useful for setting dietary and health counseling for MetS patients, especially those with low HDL-c levels. The use of a consensus methodology and polyphenol database might facilitate this in future studies. Future large-scale clinical trials are needed to clarify the underlying mechanisms of action and establish safe doses for the potential health effects described.

## 5. Conclusions

This study provides detailed information about the relationship between polyphenol intake and the components of MetS in a population of overweight or obese adults. Higher intake of all the subclasses of polyphenols was associated with a better profile of the components of MetS, especially with HDL-c levels.

## Figures and Tables

**Table 1:** Baseline characteristic of participants by quartiles of total polyphenol intake.

	Q1 (<623, 3 mg/d)	Q2 (623.4–799.4 mg/d)	Q3 (799.5–1019.2 mg/d)	Q4 (>1019.3 mg/d)	*p*	*p for Linear Trend*
***n***	1658	1658	1660	1657		
Age, years	65.2 ± 4.90	64.8 ± 4.87	65.0 ± 4.87	64.9 ± 4.98	0.10	0.19
Women, *n (%)*	894 (53.9)	845 (51.0)	785 (47.3)	685 (41.3)	<0.001	<0.001
Family history of CVD ^1^, *n (%)*	659 (39.7)	698 (42.1)	662 (39.9)	678 (40.9)	0.48	0.81
Current smokers, *n (%)*	197 (11.9)	205 (12.4)	203 (12.2)	216 (13.0)	0.78	0.36
Former smokers, *n (%)*	647 (39.0)	695 (41.9)	728 (43.9)	800 (48.3)	<0.001	<0.001
BMI, kg/m^2^	32.6 ± 3.46	32.6 ± 3.49	32.6 ± 3.51	32.3 ± 3.31	0.03	0.02
Waist circumference, cm	107.0 ± 9.76	107.4 ± 9.70	107.8 ± 9.75	107.8 ± 9.36	0.06	0.01
Body weight, kg	85.2 ± 12.8	86.2 ± 12.8	87.3 ± 13.3	87.5 ± 12.8	<0.001	<0.001
Glucose, mg/dL	113.4 ± 28.9	113.9 ± 31.0	113.9 ± 29.0	113.0 ± 27.6	0.78	0.71
Glycated hemoglobin, %	6.10 ± 0.88	6.22 ± 2.58	6.25 ± 3.53	6.10 ± 0.88	0.15	0.85
Total-cholesterol, mg/dL	196 ± 38.4	197 ± 37.7	196 ± 37.0	198 ± 42.8	0.59	0.57
HDL-cholesterol, mg/dL	47.6 ± 11.5	48.2 ± 11.7	48.7 ± 12.2	47.9 ± 11.9	0.06	0.32
**Medications, *n (%)***						
Antihypertensive agents	1272 (76.7)	1285 (77.5)	1294 (77.9)	1304 (78.7)	0.48	0.46
Colesterol-lowering agents	862 (52.0)	846 (51.0)	858 (51.7)	842 (50.8)	0.97	0.52
Insulin	84 (5.07)	98 (5.91)	67 (4.04)	63 (3.80)	0.01	0.01
Metformin	380 (22.9)	404 (24.4)	383 (23.1)	347 (20.9)	0.13	0.12
Other hypoglycemic drugs	324 (19.5)	331 (20.0)	327 (19.7)	303 (18.3)	0.62	0.35
Aspirin or antiplatelet drugs	246 (14.8)	272 (16.4)	249 (15.0)	271 (16.3)	0.26	0.61
NSAIDS	534 (32.2)	469 (28.3)	484 (29.2)	446 (26.9)	0.01	0.01
Vitamins and minerals	210 (12.7)	184 (11.1)	220 (13.3)	183 (11.0)	0.19	0.11
Sedative or tranquilliser agents	417 (25.1)	416 (25.1)	389 (23.4)	392 (23.7)	0.85	0.31
Hormonal treatment (only women)	42 (2.53)	41 (2.47)	33 (1.99)	38 (2.29)	0.924	0.935
**Educational level, *n (%)***					<0.001	<0.001
Primary school	887 (53.6)	854 (51.5)	805 (48.5)	719 (43.4)		
Secondary school	468 (28.3)	467 (28.2)	497 (30.0)	481 (29.0)		
University and other studies	301 (18.2)	337 (20.3)	356 (21.5)	456 (27.5)		

^1^ Cardiovascular diseases (CVD), body mass index (BMI), high-density lipoprotein-cholesterol (HDL-c) and nonsteroidal anti-inflammatory drugs (NSAIDs). Continue variables are expressed as mean (± SD). Categorical variables are expressed as number (*n*) and percentage (%). Comparisons among quartiles of dietary polyphenol intake with Pearson’s chi square test for categorical variables or one-way ANOVA for continuous variables. For glycated hemoglobine parameter, 9% of participants had no values available. The P value for linear trend was computed by fitting a continuous variable that assigned the median value for each quartile in regression models.

**Table 2:** Contribution (%) of polyphenol subclasses to total polyphenol intake and food sources.

Polyphenol Subclasses	Contribution, Mean (mg/d) ± SD, (%)	Polyphenol Contribution as Aglycones, Mean (mg/d) ± SD, (%)	Food Sources (% of Contribution)
Total polyphenols	846 ± 318	620.9 ± 273.5	
Flavonoids	491 ± 253, (58.0)	406.3 ± 237.2 (65.44)	
▪ Anthocyanins	43.5 ± 37.8, (5.14)	24.7 ± 21.7 (3.98)	Cherries (42.2), red wine (24.1), olives (10.5), strawberries (10.1), grape (9.30), other foods (3.8)
▪ Chalcones	0.009 ± 0.18, (<0.01)	0.006 ± 0.01 (<0.01)	Beer (100)
▪ Dihydrochalcones	1.72 ± 1.59, (0.20)	0.98 ± 0.91 (0.16)	Apples (93.2), fruit juices from concentrate (6.77)
▪ Dihydroflavonols	2.62 ± 4.92, (0.31)	1.81 ± 3.43 (0.29)	Red wine (97.6), white wine (1.80), rosé wine (0.59)
▪ Catechines	28.1 ± 22.4, (3.32)	27.1 ± 20.7 (4.36)	Tea (23.0), red wine (19.2), apples (18.6), chocolate (11.6), peaches (6.0), cocoa powder (3.18), fruit juices from concentrate (2.83), other foods (15.6)
▪ Proanthocyanidins	204± 185, (24.1)	200.7 ± 189.4 (32.32)	Chocolate (42.7), apples (20.4), plums (9.53), red wine (7.09), cocoa powder (5.68), strawberries (4.20), other foods (10.4)
▪ Theaflavin	0.70 ± 1.81, (0.08)	0.57 ± 1.46 (0.09)	Tea (100)
▪ Flavanones	83.2 ± 76.6, (9.83)	58.1 ± 55.0 (9.35)	Oranges (71.3), natural orange juice (23.0), fruit juices from concentrate (3.22), other foods (2.09)
▪ Flavones	73.2 ± 47.4, (8.65)	54.7 ± 32.9 (8.81)	Whole-grain bread (30.0), bread (23.6), oranges (21.6), natural orange juice (8.53), artichoke (3.80), other foods (12.5).
▪ Flavonols	54.0 ± 22.3, (6.40)	35.6 ± 15.3 (5.73)	Onions (27.8), spinach (26.7), lettuce (11.9), red wine (6.02), olives (5.10), asparagus (4.93), other foods (17.55)
▪ Isoflavonoids	0.002 ± 0.004, (<0.01)	0.002 ± 0.003 (<0.01)	Beer (100)
Phenolic acids	280 ± 131, (33.1)	164.2 ± 70.8 (26.44)	
▪ Hydroxybenzoic acids	15.5 ± 10.3, (1.83)	20.5 ± 12.4 (3.30)	Red wine (21.2), olives (19.9), walnuts (18.1), tea (9.46), swiss chard leaves (6.15), white wine (1.34), other foods (23.8)
▪ Hydroxycinnamic acids	264 ± 129, (30.9)	141.6 ± 66.8 (22.80)	Decaffeinated coffee (37.7), coffee (26.1), plums (5.66), potatoes (5.50), olives (4.21), red wine (1.79), other foods (19.0)
▪ Hydroxyphenylacetic acids	0.90 ± 1.04, (0.10)	1.16 ± 1.40 (0.19)	Olives (87.2), red wine (6.57), beer (3.86), extra virgin olive oil (1.52), white wine (0.65)
▪ Hydroxyphenylpropanoic acids	0.48 ± 0.65, (0.06)	0.91 ± 1.23 (0.14)	Olives (100)
Stilbenes	2.13 ± 3.92, (0.25)	1.78 ± 3.19 (0.29)	Red wine (91.9), white wine (3.94), grapes (1.60), rosé wine (1.21), other foods (0.07)
Lignans	1.53 ± 0.56, (0.18)	1.33 ± 0.55 (0.21)	Extra virgin olive oil (16.7), seeds (9.84), oranges (9.73), green bean (5.42), pepper (5.32), peaches (4.97), broccoli (4.71), bread (4.48), red wine (4.16), cabbage (2.77), other foods (31.9)
Other polyphenols	70.8 ± 41.5, (8.37)	45.6 ± 27.8 (7.34)	
▪ Alkylmethoxyphenols	0.93 ± 0.87, (0.11)	0.93 ± 0.87 (0.15)	Decaffeinated coffee (74.1), coffee (16.2), beers (9.77)
▪ Alkylphenols	13.7 ± 17.8, (1.62)	13.8 ± 18.5 (2.23)	Whole-grain bread (69.1), whole-grain pastries (14.8), breakfast cereals (8.40), pasta (3.29), other foods (4.41)
▪ Furanocoumarins	0.37 ± 0.38, (0.04)	0.37 ± 0.39 (0.06)	Celery stalks (98.3), grapefruit juice (1.7)
▪ Hydroxybenzaldehydes	0.42 ± 0.65, (0.05)	0.42 ± 0.66 (<0.01)	Red wine (78.9), walnuts (14.5), beer (2.61), white wine (1.95), other foods (2.04)
▪ Hydroxybenzoketones	0.002 ± 0.004, (<0.01)	0.002 ± 0.003 (<0.01)	Beer (100)
▪ Hydroxycoumarins	0.10 ± 0.19, (0.01)	0.09 ±0.18 (<0.01)	Beer (73.6), white wine (26.3), cocoa powder (0.10)
▪ Methoxyphenols	0.13 ± 0.12, (0.01)	0.11 ± 0.12 (0.01)	Decaffeinated coffee (81.3), coffee (18.7)
▪ Naphtoquinones	0.82 ± 1.12, (0.09)	0.84 ± 1.14 (0.14)	Walnuts (100)
▪ Tyrosols	52.4 ± 37.8, (6.19)	30.0 ± 21.2 (4.83)	Olives (50.0), extra virgin olive oil (34.8), refined olive oil (5.17), red wine (3.29), other foods (6.74)
▪ Other	1.96 ± 2.30, (0.23)	0.66 ± 0.54 (0.11)	Orange juice (45.4), pears (18.2), coffee (16.0), other fruit juices (9.98), olives (5.86), other foods (4.56)

**Table 3:** Energy-adjusted intake of total polyphenol and their main subclasses according to sociodemographic and lifestyle characteristics.

	*n*	Total Polypenols (mg/d)	*p*	Flavonoids (mg/d)	*p*	Phenolic Acids (mg/d)	*p*	Stilbenes (mg/d)	*p*	Lignans (mg/d)	*p*	Other Polyphenols (mg/d)	*p*
Total population	6633	846 ± 275 ^1^		491 ± 229		290 ± 127		2.13 ± 3.81		1.53 ± 0.54		70.8 ± 38.5	
Men	3424	830 ± 288	<0.001	469 ± 234	<0.001	285 ± 134	0.003	3.00 ± 4.74	<0.001	1.53 ± 0.54	0.933	72.1 ± 42.5	0.006
Women	3209	863 ± 259		515 ± 220		276 ± 118		1.21 ± 2.12		1.53 ± 0.53		69.5 ± 33.7	
Age (years)													
<65	3530	835 ± 275	0.002	476 ± 230	<0.001	285 ± 128	0.014	2.15 ± 4.03	0.605	1.51 ± 0.54	0.006	70.7 ± 39.2	0.967
65-70	2122	854 ± 271		503 ± 225		276 ± 123		2.07 ± 3.62		1.55 ± 0.52		71.0 ± 38.3	
>70	981	866 ± 281		517 ± 228		275 ± 127		2.21 ± 3.40		1.55 ± 0.54		70.8 ± 36.4	
BMI (Kg/m^2^)													
<29.9	1762	847 ± 268	0.042	501 ± 225	0.004	272 ± 124	0.006	2.26 ± 3.85	<0.001	1.52 ± 0.49	0.679	69.9 ± 36.8	0.353
30-34.9	3258	852 ± 280		493 ± 232		284 ± 129		2.24 ± 3.90		1.53 ± 0.54		71.5 ± 39.7	
>35	1613	831 ± 270		475 ± 226		282 ± 124		1.77 ± 3.57		1.54 ± 0.57		70.5 ± 37.9	
Physical activity level													
Low	3953	833 ± 278	<0.001	480 ± 231	<0.001	280 ± 129	0.884	1.85 ± 3.48	<0.001	1.51 ± 0.54	<0.001	70.0 ± 38.5	0.034
Moderate	1253	861 ± 267		503 ± 217		282 ± 123		2.30 ± 3.79		1.55 ± 0.54		71.7 ± 36.6	
Active	1408	867 ± 271		510 ± 230		280 ± 123		2.76 ± 4.55		1.58 ± 0.53		72.8 ± 40.0	
Educational level													
Primary school	3266	834 ± 259	<0.001	482 ± 213	<0.001	278 ± 121	0.070	1.80 ± 3.38	<0.001	1.54 ± 0.55	0.093	70.9 ± 40.2	0.290
Secondary school	1913	840 ± 270		487 ± 227		279 ± 125		2.27 ± 3.98		1.51 ± 0.53		69.9 ± 38.1	
University	1450	880 ± 311		517 ± 260		287 ± 139		2.70 ± 4.40		1.55 ± 0.52		72.0 ± 35.0	
Smoking status													
Current smokers	821	841 ± 296	0.581	455 ± 243	<0.001	311 ± 143	<0.001	2.33 ± 4.43	0.114	1.47±0.53	<0.001	70.5 ± 46.3	0.768
Non-smokers	5812	847 ± 272		496 ± 226		276 ± 123		2.10 ± 3.72		1.54±0.54		70.9 ± 37.3	

^1^ Mean ± Standard deviation. BMI: body mass index. Total and polyphenol subclasses were adjusted for total energy intake using the residual method. Comparison between subcategories was performed using ANOVA.

## Supplementary Materials

The following are available online at https://www.mdpi.com/2072-6643/12/3/689/s1, Table S1: Energy-adjusted sub-classes of dietary polyphenol intake by metabolic syndrome criterias.
