# Supplementary material for: Dietary Polyphenol Intake is Associated with HDL-Cholesterol and A Better Profile of other Components of the Metabolic Syndrome: A PREDIMED-Plus Sub-Study
Source: Nutrients. 2020 Mar 4;12(3):689. doi: 10.3390/nu12030689 (PMC7146338; doi:10.3390/nu12030689)
Supplement: Supplementary file 1 [file nutrients-12-00689-s001.pdf]

Supplementary table 1: Energy-adjusted sub-classes of dietary polyphenol intake by metabolic syndrome criterias

	Waist circumference, *cm*		Systolic blood pressure, *mmHg*		Diastolic blood pressure, *mmHg*		Fasting plasma glucose, *mg/dL*		HDL-cholesterol, *mg/dL*		Triglycerides, *mg/dL*	
	**β**	**p-value**	**β**	**p-value**	**β**	**p-value**	**β**	**p-value**	**β**	**p-value**	**β**	**p-value**
**Flavonoids**[1]												
**Model 1**[*]	-0.44	<0.001	-0.07	0.71	0.31	0.01	-1.00	0.002	0.33	0.01	-1.59	0.06
**Model 2**[**]	-0.32	0.001	-0.03	0.86	0.32	0.01	-0.44	0.11	0.27	0.03	-1.11	0.20
**Model 3**[***]	-0.23	0.02	-0.15	0.43	0.22	0.06	-0.49	0.08	0.25	0.04	-1.23	0.17
**Phenolic acids**												
**Model 1**	0.32	0.001	-0.11	0.54	-0.21	0.06	1.55	<0.001	0.10	0.46	-0.87	0.31
**Model 2**	0.32	0.001	-0.15	0.43	-0.20	0.08	0.53	0.06	0.14	0.26	-1.27	0.14
**Model 3**	0.36	<0.001	-0.10	0.60	-0.23	0.05	0.77	0.007	0.15	0.23	-1.37	0.11
**Stilbenes**												
**Model 1**	-0.37	<0.001	0.31	0.09	0.12	0.29	-0.08	0.81	1.11	<0.001	-2.46	0.005
**Model 2**	-0.22	0.03	0.35	0.06	0.15	0.20	-0.04	0.90	1.10	<0.001	-2.11	0.01
**Model 3**	-0.17	0.11	0.39	0.06	0.25	0.05	0.03	0.90	1.42	<0.001	-2.63	0.006
**Lignans**												
**Model 1**	-0.02	0.83	0.45	0.01	0.63	<0.001	-1.03	0.001	0.38	0.002	-2.25	0.01
**Model 2**	0.08	0.43	0.43	0.02	0.63	<0.001	-0.52	0.06	0.34	0.005	-1.85	0.03
**Model 3**	0.18	0.09	0.32	0.11	0.46	<0.001	-0.63	0.04	0.24	0.07	-2.76	0.004
**Other polyphenols**												
**Model 1**	-0.08	0.44	-0.82	<0.001	-0.41	<0.001	0.24	0.46	0.40	0.001	-0.04	0.96
**Model 2**	-0.03	0.75	-0.80	<0.001	-0.40	<0.001	-0.08	0.78	0.39	0.001	0.05	0.95
**Model 3**	-0.03	0.76	-0.65	0.001	-0.37	0.002	-0.17	0.53	0.38	0.001	0.18	0.83
**Total polyphenols**												
**Model 1**	-0.03	0.77	0.01	0.96	0.23	0.05	-0.34	0.29	0.48	<0.001	-1.19	0.17
**Model 2**	0.04	0.69	0.04	0.81	0.24	0.04	0.08	0.78	0.47	<0.001	-1.08	0.21

**Model 3**	0.02	0.85	-0.03	0.90	0.07	0.63	0.07	0.83	0.37	0.007	-1.91	0.05

Lineal regression model: Differences among MetS criteria were evaluated using lineal models based on mean differences. We used robust variance estimators to account for intracluster correlation in all lineal models, considering members of the same household as a clusters.

[1] Polyphenol and polyphenol subclasses are expressed as quartiles of energy-adjusted dietary intake. Therefore, the β coefficient is compared to the Q1 of intake.

*__Model 1:__ adjusted for sex, age (three categories; >70 years; 65-70 years or <65 years), recruiting center, and robust variance estimators.

**__Model 2__: Model 1 + physical activity level (three categories; low, moderate, high), BMI (three categories; <30, 30-35, >35 kg/m^2, except for waist circumference criteria), smoking status (three categories; never smoker, smoker and current smoker) and educational level (three categories; primary, secondary school and University). For the analysis of glycemia we adjusted for anti-diabetic drugs and systolic and diastolic blood pressure was adjusted for anti-hipertensive drug treatments.

***__Model 3__: Model 2 + energy intake (continous, kcal/d), saturated fatty acids (g/d), and alcohol intake from distillated drinks (g/d). For the analysis of glycemia we adjusted for dietary simple sugars intake (g/d) and for the analysis of systolic and diastolic blood pressure was adjusted for dietary sodium intake (mg/d).
